# Bacterial challenge-associated metabolic phenotypes in *Hermetia illucens* defining nutritional and functional benefits

**DOI:** 10.1038/s41598-021-02752-8

**Published:** 2021-12-02

**Authors:** Phuc N. Ho, Poramate Klanrit, Yupa Hanboonsong, Umaporn Yordpratum, Manida Suksawat, Thanaporn Kulthawatsiri, Anyarin Jirahiranpat, Suthicha Deewai, Panya Mackawan, Rasana W. Sermswan, Nisana Namwat, Watcharin Loilome, Tueanjit Khampitak, Arporn Wangwiwatsin, Jutarop Phetcharaburanin

**Affiliations:** 1 Department of Biochemistry, Faculty of Medicine, Khon Kaen University, Khon Kaen, 40002 Thailand; 2 Khon Kaen University International Phenome Laboratory, Khon Kaen, 40002 Thailand; 3 Cholangiocarcinoma Research Institute, Khon Kaen University, Khon Kaen, 40002 Thailand; 4 Department of Entomology and Plant Pathology, Faculty of Agriculture, Khon Kaen University, Khon Kaen, 40002 Thailand; 5 Department of Microbiology, Faculty of Medicine, Khon Kaen University, Khon Kaen, 40002 Thailand; 6 Research and Development Center, Betagro Group, Klong Luang, Pathum Thani 12120 Thailand; 7 Center of Excellence for Innovation in Chemistry, Faculty of Science, Khon Kaen University, Khon Kaen, 40002 Thailand

**Keywords:** Metabolomics, Entomology

## Abstract

Black soldier fly (BSF, *Hermetia illucens*) is popular for its applications in animal feed, waste management and antimicrobial peptide source. The major advantages of BSF larva include their robust immune system and high nutritional content that can be further developed into more potential agricultural and medical applications. Several strategies are now being developed to exploit their fullest capabilities and one of these is the immunity modulation using bacterial challenges. The mechanism underlying metabolic responses of BSF to different bacteria has, however, remained unclear. In the current study, entometabolomics was employed to investigate the metabolic phenoconversion in response to either *Escherichia coli*, *Staphylococcus aureus*, or combined challenges in BSF larva. We have, thus far, characterised 37 metabolites in BSF larva challenged with different bacteria with the major biochemical groups consisting of amino acids, organic acids, and sugars. The distinct defense mechanism-specific metabolic phenotypes were clearly observed. The combined challenge contributed to the most significant metabolic phenoconversion in BSF larva with the dominant metabolic phenotypes induced by *S. aureus*. Our study suggested that the accumulation of energy-related metabolites provided by amino acid catabolism is the principal metabolic pathway regulating the defense mechanism. Therefore, combined challenge is strongly recommended for raising BSF immunity as it remarkably triggered amino acid metabolisms including arginine and proline metabolism and alanine, aspartate and glutamate metabolism along with purine metabolism and pyruvate metabolism that potentially result in the production of various nutritional and functional metabolites.

## Introduction

Insect has been widely used nowadays in the world as a sustainable food source with high nutrient values. The black soldier fly (BSF), *Hermetia illucens* (Diptera: Stratiomyidae), is one the most essential insects for their values in animal feed, bioconversion, and biological active molecules (antimicrobial peptides, chitin, lipids, and odorant binding proteins)^1–6^. BSF has been reared by many companies on an industrial scale with the number up to a thousand kilograms per week or month, especially those in Netherlands and United Kingdom with an average funding from investors of more than 100 million USD^7,8^. The larval stage of BSF provides not only a high protein content (from 37 to 63% of their dry matter), but also other macro- and micronutrients such as fat, chitin, vitamins, and minerals^9^. Compared to common protein-rich feeds (soya bean meal, fish meal, *etc*.), BSF contains high levels of some essential amino acids such as lysine, methionine, and threonine and demonstrates a better amino acid profile^10^. With this valuable content of nutrition, BSF is believed to bring several significant benefits for human consumption thus discussing on food safety concerns, functional properties, and biomolecule fractionation protocol is gaining more attention^11,12^. Moreover, another remarkable potential of BSF is antimicrobial property which is a promising approach to cope with the multidrug-resistant bacteria crisis. A transcriptome study of BSF has identified several antimicrobial peptide (AMP) sequences with putative anticancer, antiviral, and antifungal functions^13^. Hence, this has strengthened the multipurpose uses of BSF to be exploited for agriculture, nutrition, as well as medicine.

Given that the robust immune system of BSF plays an important role in improvement of feeding and bioactive compound synthesis, bacterially-challenged immunity is one of the main approaches used nowadays^7^. Different bacterial strains were applied to trigger biological processes in BSF larvae in studying nutritional immunology, purification, and characterisation of novel bioactive compounds such as AMPs and fatty acids^13–19^. The results, notably, showed the enhanced antimicrobial abilities against various strains of Gram-negative^13,14,16–18^ and Gram-positive bacteria^15–17^, fungi^16^, and proposed potentials of antiviral and anticancer properties^13,20^. However, none of the studies have, so far, discussed the metabolic phenoconversion of BSF to different kinds of bacterial challenges.

Entometabolomics, the metabolic profiling application in insect study, is the powerful analytical measurement of cellular interaction and intercorrelation of insect metabolites^21^. Metabolomic analysis in studies of *Drosophila*, a classical model organism, was successfully applied in not only physiology aspects of invertebrates but also in disease model and drug treatments^22–24^. Thus, in the current study, entometabolomics has been applied to characterise the alteration of biochemical components in BSF larva and to investigate the metabolic phenotypes in response to different bacterial challenges that may result in various biomolecule pools for further agricultural and medical uses.

## Results

### Biochemical characterisation of BSF using entometabolomics

**Figure 1:**
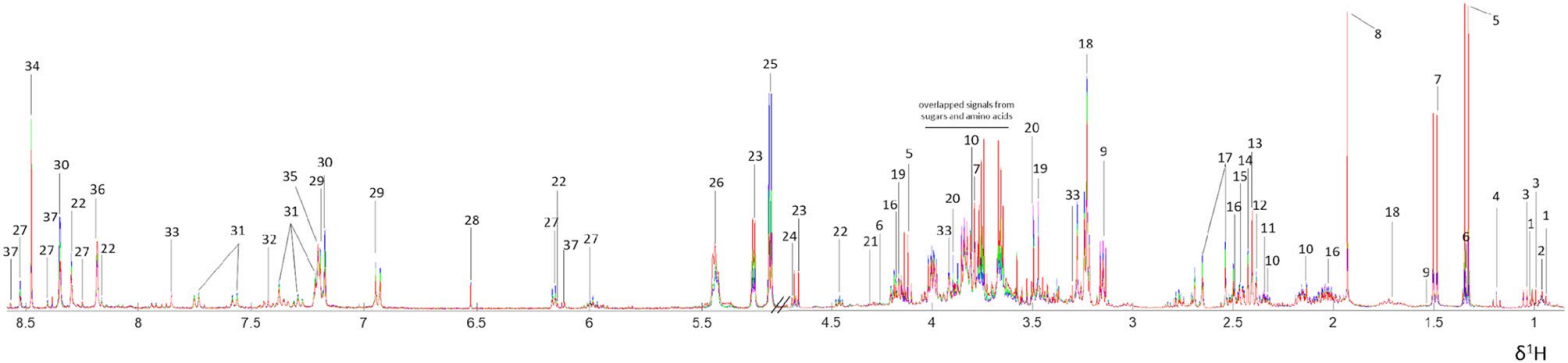
Representative median ^1^H NMR CPMG spectra. BSF larva unchallenged control (purple), BSF larva challenged with single *E. coli* (blue), single *S. aureus* (green), and combined *E. coli* and *S. aureus* (red). Key: 1, isoleucine; 2, leucine; 3, valine; 4, ethanol; 5, lactate; 6, threonine; 7, alanine; 8, acetate; 9, lysine; 10, glutamate; 11, thymidine; 12, oxaloacetate; 13, succinate; 14, 2-oxoglutarate; 15, carnitine; 16, pyroglutamate; 17, citrate; 18, putrescine; 19, choline; 20, betaine; 21, malate; 22, guanosine; 23, glucose; 24, glucose-6-phosphate; 25, trehalose; 26, maltose; 27, NADPH (nicotinamide adenine dinucleotide phosphate); 28, fumarate; 29, tyramine; 30, histamine; 31, indoxyl sulfate; 32, phenylalanine; 33, dimethylxanthine; 34, formate; 35, syringic acid; 36, *N*-formylglycine; 37, IMP (inosine monophosphate). The figure was plotted using MATLAB R2021a version 9.10 (MathWorks, Inc., USA).

To investigate biochemical components in BSF, larval extracts from four different groups were analysed using nuclear magnetic resonance (NMR) spectroscopy-based metabolomics. Spectral data was then acquired and pre-processed. With the median ^1^H NMR CPMG spectra, a total of 37 metabolites were identified in BSF larval extracts challenged with either single or combined bacteria and unchallenged control (Fig. 1). Two-dimensional (2D) NMR experiments were also conducted to aid the metabolite assignment. Full list of identified metabolites can be found in Supporting Information (Table S1). The major groups of metabolites were amino acids, organic acids, and sugars which caused strong overlapped signals within the region from approximately δ^1^H 3.5 to 4.0.

### Bacterial challenge-induced metabolic responses in BSF

**Figure 2:**
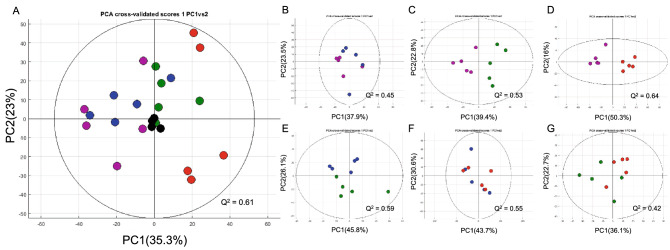
Overview of metabolic similarities and differences observed in BSF control and BSF post-bacterial challenges. PCA cross-validated score plots of (**A**) all classes, (**B**) control versus single *E. coli* challenge, (**C**) control versus single *S. aureus* challenge, (**D**) control versus combined challenge, (**E**) single *E. coli* challenge versus single *S. aureus* challenge, (**F**) single *E. coli* challenge versus combined challenge and (**G**) single *S. aureus* challenge versus combined challenge. Single *E. coli* challenge, blue; single *S. aureus* challenge, green; combined challenge, red; unchallenged control, purple; quality control (QC), black. The figures were plotted using MATLAB R2021a version 9.10 (MathWorks, Inc., USA).

To investigate the metabolic overview, similarities, and differences of all classes, principal component analysis (PCA) was performed. The PCA cross-validated score plot shows a tight clustering of quality control (QC) samples indicating no analytical variation and high analytical precision (Fig. 2A, R^2^: PC1 = 35.3% and PC2 = 23.0%; Q^2^ = 0.61). Moreover, single *S. aureus* and combined challenge groups were differentiated from the single *E. coli* challenge group and unchallenged control along the first principal component (PC1) (Fig. 2A). To maximise the meaningfulness of metabolome data, six pairwise PCA models were constructed. Pairwise PCA cross-validated score plots show the altered metabolic profiles of BSF larva challenged with either single *E. coli* (Fig. 2B, R^2^: PC1 = 37.9% and PC2 = 23.5%; Q^2^ = 0.45), single *S. aureus* (Fig. 2C, R^2^: PC1 = 39.4% and PC2 = 22.8%; Q^2^ = 0.53) or combined (Fig. 2D, R^2^: PC1 = 50.3% and PC2 = 21.6%; Q^2^ = 0.64) bacterial injection compared with unchallenged control. Metabolic differences between BSF larva challenged with single *E. coli* and single *S. aureus* were observed along the second principal component (PC2) (Fig. 2E, R^2^: PC1 = 45.8% and PC2 = 26.1%; Q^2^ = 0.59). Interestingly, no metabolic differences were observed when comparing single *E. coli* challenge and combined challenge groups (Fig. 2F, R^2^: PC1 = 43.7% and PC2 = 30.6%; Q^2^ = 0.55), while clearer class separation was detected between single *S. aureus* and combined challenge groups (Fig. 2G, R^2^: PC1 = 36.1% and PC2 = 22.7%; Q^2^ = 0.42).

**Figure 3:**
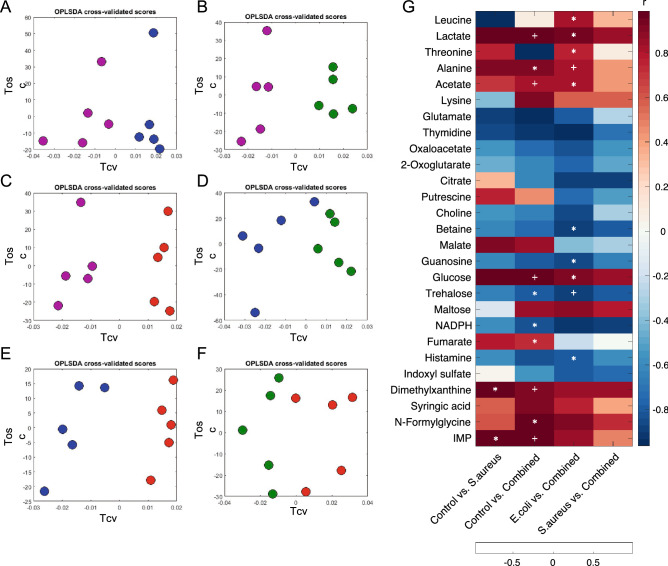
Impact of the different bacterial challenges on BSF metabolic responses. O-PLS-DA score plots of (**A**) control versus single *E. coli* challenge, (**B**) control versus single *S. aureus* challenge, (**C**) control versus combined challenge, (**D**) single *E. coli* challenge versus single *S. aureus* challenge, (**E**) single *E. coli* challenge versus combined challenge, and (**F**) single *S. aureus* challenge versus combined challenge. (**G**) Correlation coefficient (r) shown in the heatmap was obtained from O-PLS-DA models. Positive (red) or negative (blue) r values indicate higher or lower concentrations of the metabolites in either group of comparison. * and + indicate significant levels of metabolite differences between pairwise groups at *p* < 0.05 and *p* < 0.01, respectively using Kruskal Wallis, Dunn *post-hoc* test with Benjamini–Hochberg correction. The figures were plotted using MATLAB R2021a version 9.10 (MathWorks, Inc., USA).

Orthogonal projection to latent structures discriminant analysis (O-PLS-DA) was performed to provide insights into specific metabolic reprogramming of BSF larva induced by different bacterial challenges. The O-PLS-DA cross-validated score plots from all pairwise comparison models show the distinct class discrimination (R^2^X ≥ 54.0%, Q^2^Y ≥ 0.67, permutation *p* = 0.01, Fig. 3A–F, Table S2). However, two models including unchallenged control versus single *E. coli* challenge and single *E. coli* challenge versus single *S. aureus* challenge did not show any significant discriminatory metabolites on O-PLS-DA corresponding loading plots following Benjamini-Hochberg false discovery rate correction despite the valid models (permutation *p*-value = 0.01) (Fig. S1A,D). Hence, the O-PLS-DA correlation coefficients of 27 significant metabolites along with the statistically significant levels obtained from Kruskal Wallis, Dunn *post-hoc* test with Benjamini-Hochberg correction of the remaining four models were summarised in the heatmap where red-blue colour intensity represents positive and negative correlation coefficients, respectively, and * and + represent the statistical significant levels, *p* < 0.05 and *p* < 0.01, respectively (Fig. 3G). BSF larva challenged with single *S. aureus* demonstrated higher levels of glycolytic metabolites (e.g*.*, glucose and lactate), tricarboxylic acid (TCA) cycle intermediates (e.g*.*, malate and fumarate), amino acid (e.g., alanine) polyamine (e.g*.*, putrescine) and organic compounds (e.g*.*, 1,7-dimethylxanthine and IMP) accompanied with decreased levels of glutamate and betaine compared with unchallenged control (Fig. 3G and S1B). Although no significant differential metabolite was observed in the single *E. coli* challenge group, combined *S. aureus* and *E. coli* challenge still exhibited the promising metabolic alterations compared with unchallenged control. Interestingly, significantly elevated metabolites found in combined challenge were similar to those observed in single *S. aureus* challenge with additional differential metabolites that include higher levels of lysine, maltose, syringic acid and *N*-formylglycine, and decreased levels of thymidine, threonine, guanosine, trehalose, NADPH and histamine (Fig. 3G and S1C). To further investigate the metabolic differences between single bacterial challenge and combined bacterial challenge, two pairwise comparison models consisting of combined challenge versus single *E. coli* challenge and combined challenge versus single *S. aureus* challenge were constructed. It was clearly seen that single *E. coli* challenge and unchallenged control showed the similar metabolic fingerprints when compared with the combined challenge (Fig. 3G and S1E), whereas single *S. aureus* challenge-specific metabolic phenotype when compared with the combined challenge, was evident by the increased levels of NADPH and citrate accompanied with decreased levels of 1,7-dimethylxanthine, glucose and maltose (Fig. 3G and S1F).

### Metabolic reprogramming occurred in BSF larva post-bacterial challenges

**Figure 4:**
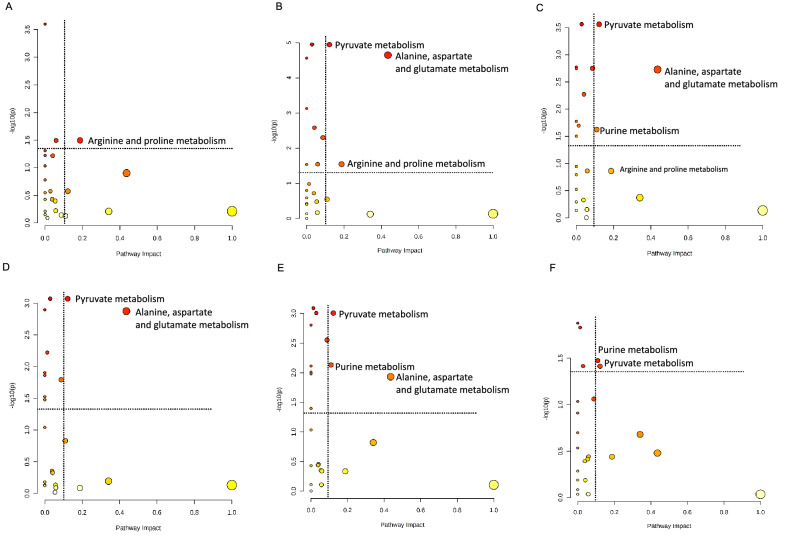
Graphical overview for the metabolome view of the pathway analysis. (**A**) control versus single *E. coli* challenge, (**B**) control versus single *S. aureus* challenge, (**C**) control versus combined challenge, (**D**) single *E. coli* challenge versus single *S. aureus* challenge, (**E**) single *E. coli* challenge versus combined challenge, and (**F**) single *S. aureus* challenge versus combined challenge. X-axis indicates the impacts on pathway, while Y-axis indicates the significance of changes (– log (*p*)) in the pathway by the identified metabolites represented by different colour intensities. Cut-off values for pathway impact score > 0.1, false discovery rate < 0.25 and *p* < 0.05. The figures were obtained from MetaboAnalyst using our experimental data as input.

In order to measure the involvement and directions of post-bacterial challenge effects on metabolic adaptation of BSF larva, pathway analysis of significantly contributed metabolites was performed using MetaboAnalyst based on KEGG pathway of insect database. The results showed a total of four metabolic pathways that had remarkable impacts including alanine, aspartate and glutamate metabolism, arginine and proline metabolism, purine metabolism, and pyruvate metabolism (Fig. 4). Apparently, metabolic reprogramming after bacterial challenges in BSF larva involved amino acid metabolism, carbohydrate metabolism, and nucleotide metabolism. Our findings also demonstrated the specific pathways corresponded to the different challenge groups. While metabolic responses in BSF larva after single *E. coli* challenge included only arginine and proline metabolism, single *S. aureus* challenge impacted the two additional pathways that are amino acid-related (alanine, aspartate and glutamate metabolism) and energy-related metabolism (pyruvate metabolism). Moreover, as shown in Fig. 4C,E,F, purine metabolism particularly showed statistically significant difference (impact score > 0.1, *p* < 0.05, FDR < 0.25) in those models involving the combined challenge.

## Discussion

BSF is one of the most essential insects for their outstanding potential in animal feed, bioconversion, and antimicrobial agent^7^. Hence, several approaches have been applied to fulfil the knowledge and to maximise the use of BSF, especially its robust immune system. However, the global metabolic profile and cellular metabolism of BSF in response to bacterial infection has remained unclear. By using ^1^H NMR spectroscopy-based metabolomics approach, our study has indicated a total of 37 metabolites from BSF larva that were bacterially-challenged and unchallenged. The major biochemical classes found in BSF included organic acids (e.g*.,* lactate, acetate, oxaloacetate, succinate, 2-oxoglutarate, pyroglutamate, citrate, malate and fumarate), fundamental amino acids (e.g*.,* isoleucine, leucine, valine, threonine, alanine, lysine, glutamate and phenylalanine), and sugars (e.g*.,* glucose, maltose and trehalose). Moreover, gut-microbiota metabolites (e.g*.,* ethanol, formate^25^ and indoxyl sulfate), conditionally-essential nutrients^26^ (e.g*.,* choline and carnitine), biogenic amines (e.g*.* putrescine, tyramine and histamine) and other amino acid derivatives (e.g*.,* betaine, NADPH and *N*-formylglycine), plant metabolite (e.g*.,* 1,7-dimethylxanthine and syringic acid), nucleoside (e.g*.,* thymidine and guanosine), and nucleotide (e.g*.,* IMP) were observed. Compared to previous metabolomic studies of other insects, BSF shared 11 metabolites similar to *Drosophila melanogaster*^22^ (Diptera), 13 metabolites to *Manduca sexta*^27^ (Lepidoptera), and 14 metabolites to *Schistocerca gregaria*^28^ (Orthoptera). The similarities, mostly from amino acids, organic acids, and sugars, have consolidated the baseline biochemical profile of BSF and other insects in general.

Although the appearance of some distinct metabolites in BSF provided an insight in their biochemical characteristics, most of the underlying mechanisms in response to different bacterial challenges, however, inquired further study. Ethanol and formate, the compounds produced by anaerobic metabolism of microorganisms, have been previously found in the gut of *Pachnoda ephippiata* larva (Coleoptera) suggesting the gut microbial fermentation in insects^29^. Various studies in BSF demonstrated that the gut microbial community is expressively involved in growth, nutrition, as well as its utilities in waste-management and animal feed^30–34^. Hence, the detection of gut microbiota metabolites in this study will help further our understanding of BSF-gut microbiota metabolic interactions. *N*-formylglycine is known as peptide derived from enzymic formylation of melittin—main component of honeybee (e.g*., Apis mellifera*) venom and antimicrobial peptide^35,36^. The bioactivities of this metabolite were further emphasised in the research of biosynthetic pathway from marine *Streptomyces* bacteria, reporting that the metabolic product, streptophenazines, with the attachment of *N*-formylglycine provides the strong antibiotic activity^37^. Thus, *N*-formylglycine could be employed in BSF to cope with bacterial infection through either AMP modification or gut microbiota metabolism. Another identified metabolite involved in defense mechanism against pathogens was 1,7-dimethylxanthine or paraxanthine, one of the well-known products of the metabolism of caffeine—a plant purine alkaloid that is employed to defend against herbivores and pathogens^38^. Paraxanthine is involved in caffeine degradation in *D. melanogaster*^39^, suggesting that it may serve a similar function in BSF following bacterial challenges, especially single *S. aureus* and combined *E. coli* and *S. aureus* challenges.

What is more, our findings depicted the unexpected metabolites of which their detections in insect still lack adequate information. Syringic acid is a common phenolic compound found in vegetables and fruits^40^. In addition, it has been defined as a metabolite of anthocyanin-related metabolism in human gut microbes^40–43^. Nevertheless, the biosynthesis and degradation of anthocyanin in insects have not been fully investigated thus far. This suggests that syringic acid found in the current study was potentially derived from the food components, brewer’s grain and palm kernel meal^44–48^ used in the current study. Indoxyl sulfate (IS), the metabolite known as uremic toxin in human, was also identified in BSF larval extracts. The production of IS begins with dietary tryptophan that can be metabolised by gastrointestinal bacteria to produce indole which is eventually absorbed through gut epithelial barrier and converted into IS in the liver by sulfotransferase Family 1A Member 1 (SULT1A1)^49–51^. Though, to the best of our knowledge, the production or related information of IS in insect has yet to be reported. The possibility could be that the intercorrelations between microbial communities and digestive enzymes in BSF gut facilitate the production of IS. In accordance with this concept, a previous study performing qualitative and quantitative enzymatic assays demonstrated the rich and robust gastric enzymes compared with housefly^52^ along with its gut microbiota function that aid several metabolic reactions in BSF^7^. Due to some certain similarities of enzymatic content and gut microbiota function to human, BSF may serve as a model to study specific metabolisms.

Multivariate analyses, including PCA and O-PLS-DA, were performed to visualise the similarities and differences of entometabolome datasets between non-challenged and bacterial-challenged BSF larval extracts. The results exhibited the valid class discrimination in four pairwise comparison models comprising of single *S. aureus* challenge versus control, combined challenge versus control, combine challenge versus single *E. coli* challenge, combined challenge versus single *S. aureus* challenge. Consistently, the correlation coefficients obtained from these O-PLS-DA models have indicated the differential metabolites that were significantly changed in their concentrations in either group of comparison**.** Our data suggests that combined challenge mostly triggered the metabolic phenoconversion of larva compared to individual bacterial strain challenge, that was evident by altered concentrations of 21 differential metabolites in larva. While single *S. aureus* challenge resulted in the metabolic changes, such a significant metabolic effect was not observed following *E. coli* challenge. This indicates that even though the responses caused by single *E. coli* challenge were not significant, its contribution to the integration with *S. aureus* provided more intense metabolic impacts in BSF larva. Also, the analyses between bacterially-challenged groups described the similar patterns, supporting the given hypotheses that the domination of *S. aureus* to the combined challenge were more powerful than *E. coli* alone. Our findings are consistent with the previous study suggesting that the insects employed the specific signalling pathways to encounter Gram-positive or Gram-negative bacterial infections^53^. Moreover, our results shed light further into the metabolic effects of BSF on the combined bacterial challenge. The clear clustering and separation between groups with quite a large degree of inter-variations in metabolite concentrations were observed in the comparison of single *E. coli* challenge versus combined challenge, whereas fewer metabolic alterations occurred between single *S. aureus* challenge versus combined challenge clearly suggesting that the metabolic effects of combined challenge mainly resulted from *S. aureus*. Over the years, studies involving the investigation of functional biomolecules and AMPs from BSF were inconsistent in the use of either single *S. aureus*, single *E. coli* or both as the immunomodulator, resulting in varying metabolic products^14–16,18,54,55^. Therefore, there has been, so far, no such scientific evidence providing the baseline understanding and appropriate guideline on the use of bacterial immunomodulators and their expected functional biomolecules that suit the specific utilisation, for example, nutritional animal feeds, prebiotics and probiotics. Here, we have reported, for the first time, the distinct BSF metabolic fingerprints that discriminate bacterial challenges as demonstrated by single *E. coli*, single *S. aureus* or combined challenge.

**Figure 5:**
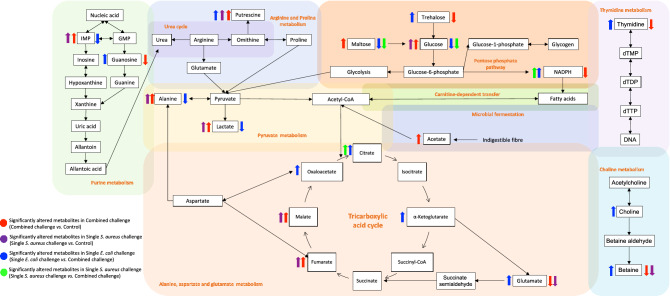
Schematic diagram of proposed altered metabolic responses to different bacterial challenges in BSF larva.

Taken together, we have summarised the proposed altered metabolic responses to different bacterial challenges in BSF larva (Fig. 5). The main metabolic pathways observed consist of purine, pyruvate, arginine and proline, alanine, aspartate and glutamate metabolism. It demonstrated that BSF immune system attempted to systemically modulate energy metabolism, amino acid metabolism, and nucleotide metabolism to defend against bacterial infection. Besides, glucose, thymidine, and choline metabolism serve the supporting information of how BSF regulates other biochemical processes to develop and maintain life-sustaining capability under circumstances of bacterial infection. Carbohydrate metabolism has been considered as the factory to supply enormous fuel for insects^56^. It employs a variety of sugars and non-carbohydrate precursors to initiate the carbohydrate synthesis^57^. Trehalose, the highly representative haemolymph sugar of insect synthesized by fat body, is also known as the main source of carbohydrate, whereas maltose is the gut-digested carbohydrate source^57^. Together with glucose, they marked the significant carbohydrate adaptation of BSF larva under microbial infection. The larva is likely to either overuse or reduce the blood sugar and exerts more of dietary carbohydrate.

Amino acids including arginine, proline, alanine, aspartate, and glutamate are all involved in mediating the intermediates of the citric acid cycle to generate energy through transamination and oxidative deamination^56^. These processes have been previously reported as the most active reaction occurring in insect fat body^58^. Particularly, amino acids are defined as one of the primary energy sources in Diptera, Orthoptera, and Coleoptera, besides lipids and carbohydrates resources^57,58^. Putrescine, another product from arginine and proline pathway, is a polyamine related to juvenile hormone specifically involved in the regulation of insect neuroblast^59^. Hence, the utilisation of amino acids by fat body to facilitate energy and protein derivative syntheses is proposed to represent BSF-defense metabolic phenotype.

In regards with nucleotide metabolism, purine metabolism plays important roles in producing uric acid for the removal of excessive ammonia and the other processes such as pigmentation, nitrogen reservation, nucleic acid synthesis, and antioxidant^60,61^. In addition, thymidine metabolism is the minor pathway of DNA synthesis promoting the cell differentiation throughout the time of insect metamorphosis^62^. Another pathway playing a crucial role in the development of insect is choline metabolism^63^. Choline is a vital nutritional metabolite for normal growth of insect. Its metabolism accommodates the production of larval haemolymph component (e.g*.,* betaine) and the regulation of nervous system metabolites (e.g*.,* acetylcholine and phosphatidylcholine)^63^. This clearly indicates that BSF larva has reprogrammed its biological processes prior to the bacterial defense mechanism to take place.

## Conclusion

The current methodology employing bacterial challenge in BSF lacks the molecular baseline information whether single or combined challenge is truly required for immunomodulation. Our study has defined the biochemical components of BSF larva with a total of 37 identified metabolites using entometabolomics. Moreover, the combined challenge exhibited the most effective immunomodulator that was evident by a large degree of metabolic phenoconversion related to energy and amino acid metabolisms. Collectively, our findings suggest the nutritional and functional benefits of combined bacterial challenge for any study involving an exploration of alternative bioactive compounds and/or AMPs from BSF larva for developing the novel agricultural and medical applications. It is noteworthy that bacterially-exposed BSF can provide additional value from nutritional and functional metabolites that suggest the alternative breeding protocol and gain greater attention from the breeders.

## Methods

### Bacterial immunisation for BSF larva

**Figure 6:**
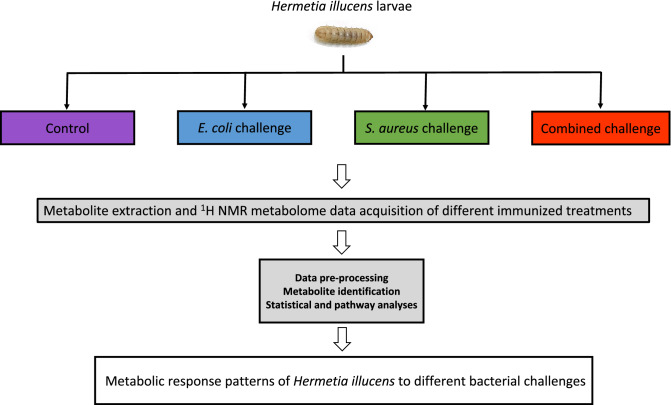
Study design and sampling to investigate metabolic phenotypes of BSF larva in response to different bacterial immunisation.

Ten-day-old larva of black soldier fly (*H. illucens*) were supplied by Industrial Insects Pilot Production Plant (Khon Kaen university, Thailand). This study has been approved by the Animal Ethics Committee of Khon Kaen University (IACUC-KKU-13/64). The five-day-old larvae were reared in the tray filled with palm kernel meal and brewer’s grain in the ratio of 5:1 and 70% moisturised with water until they reached the age of 10 days under 12 h/12 h light-dark cycle at 30–32 °C. A total of 80 larvae were disinfected in 75% alcohol for 30 min then washed with sterile water three times. The immunisation process were conducted as described previously by Alvarez et al (2019)^18^. To challenge BSF immune system, 20 larvae were individually injected using insulin needle with either *E. coli*, *S. aureus* or combined bacterial strains (1 × 10^3^ CFU/g, 10 µl) while control larvae were injected with 10 µl phosphate-buffered saline. Each challenge group included five replicates in which each replicate contained four larvae (Fig. 6). After 30 min of observation, the larvae were then transferred to new trays with the same food components and rearing method as described above for 36 h. Less than 5% of mortality was observed following 36 h. All larvae of each challenge group were then cleaned separately with sterile water for three times and were kept at − 20 °C prior to sample preparation for further analysis.

### Sample preparation for NMR spectroscopic analysis

Each replicate was weighed out and ground thoroughly in methanol:chloroform:water (1:1:0.7, v/v/v). The volume of the extraction solvent was adjusted according to weight of the sample (1 mL of solvent / 100 mg of sample). The upper aqueous phase was collected after centrifugation at 1000 g at 4 °C for 15 min and further evaporated using a speed vacuum concentrator (Labconco, MO, USA) at 40 °C until dry. The crude extracts were stored at − 80 °C prior to analysis. A total of 600 ﻿μl buffer containing 100 mM sodium phosphate, pH 7.4 in D_2_O, 0.1 mM 3-trimethysilypropionic acid (TSP) (Cambridge Isotype Laboratories, Tewksbury, MA, USA) as a chemical shift reference (δ^1^H = 0 ppm) and optionally 0.2% NaN_3_ was added to dissolve the sample. Mixture was sonicated using ultrasonicator (JeKen, China) for 10 min and filtered through 0.20 μm filter (Corning, USA) before centrifugation at 12,000 g at 4 °C for 5 min. An equal amount of 30 μl was aliquoted from all samples and pooled for the QC. Then, a total of 550 μl of supernatant was transferred into NMR tube for metabolic profiling. ﻿Proton NMR spectra were acquired using a 400 MHz NMR spectrometer (Bruker, USA) with CryoProbe Prodigy and Carr−Purcell−Meiboom−Gill (CPMG) pulse sequence [RD−90˚−(τ−180˚−τ)n−acquisition] was applied to analyse the samples at 310 K in 64 scans.

### NMR spectral data pre-processing and metabolite identification

Chemical shift referencing, baseline correction and phasing of NMR spectra were performed in TopSpin (Bruker, USA). ﻿NMR spectral data were processed using MATLAB R2021a version 9.10 (MathWorks Inc., USA) software equipped with IMPaCTS toolbox (https://doi.org/10.5281/zenodo.3077413) to conduct probabilistic quotient normalisation (PQN). Statistical total correlation spectroscopy (STOCY)^64^ was used to verify the appearances of correlated resonances on 1-dimensional NMR spectra which were searched against public databases including human metabolome database (HMDB)^65–68^ and ChenomxNMR Suite version 9.0 (Chenomx Inc., Canada). To further confirm the metabolite assignment, two-dimensional (2D) NMR experiments including correlation spectroscopy (COSY), total correlation spectroscopy (TOCSY), heteronuclear single quantum coherence (HSQC) and heteronuclear multiple bond correlation (HMBC) were performed. Data can be accessed at Open Science Framework (https://osf.io/w9tx7/).

### Statistical analysis

Multivariate statistical analysis in this study was performed in MATLAB 2021a version 9.10 environment equipped with IMPaCTS toolbox (https://doi.org/10.5281/zenodo.3077413). Pre-processed spectral data were imported into MATLAB environment for conducting principal component analysis (PCA) and orthogonal signal correction-projection to latent structures discriminant analysis (O-PLS-DA) with a pareto scaling method. All O-PLS-DA models in this study were constructed based on one predictive component and one orthogonal component. The O-PLS-DA scores and coefficient plots were also obtained. The correlation coefficient values |r| were represented through red-blue colour spectrum, where red colour indicates higher correlation while blue colour indicates lower correlation of the variables. Benjamini-Hochberg false discovery rate correction was also applied for differential biomarker discovery. ﻿The goodness of fit and predictability of the models were determined by R^2^ and Q^2^ values, respectively. The validation of all models involved in this study was assessed using cross-validation and permutation *p*-value (*p* < 0.05). Maximum intensity of selected discriminatory metabolites from valid O-PLS-DA models were calculated for quantification of absolute concentration in regards with maximum intensity and concentration of TSP. The univariate analysis was also performed in GraphPad Prism version 9.2.0 for MacOS (GraphPad Software, USA) using Kruskal Wallis, Dunn *post-hoc* test with Benjamini-Hochberg correction.

### Pathway analysis

Absolute concentrations of selected metabolites were further used for pathway impact analysis using MetaboAnalyst^69^ based on KEGG pathway library of insect^70–72^ (model organism—*Drosophila melanogaster*) to investigate the impact of different bacterial challenges on BSF metabolism. The cut-off values of 0.1 for pathway impact score, *p* < 0.05 and false discovery rates < 0.25 were applied to cover the confidence of practicable and to filter insignificant pathways^73^.

## Supplementary Information

Supplementary Information.
