# Supplementary material for: Bacterial challenge-associated metabolic phenotypes in Hermetia illucens defining nutritional and functional benefits
Source: Sci Rep. 2021 Dec 2;11:23316. doi: 10.1038/s41598-021-02752-8 (PMC8639782; doi:10.1038/s41598-021-02752-8)
Supplement: Supplementary file 1 — Supplementary Information. [file 41598_2021_2752_MOESM1_ESM.pdf]

Supporting Information for

Bacterial challenge-associated metabolic phenotypes in *Hermetia illucens* defining nutritional and functional benefits

Phuc N. Ho[1], Poramate Klanrit[1,2,3], Yupa Hanboonsong[4], Umaporn Yordpratum[5], Manida Suksawat[2,3], Thanaporn Kulthawatsiri[2,3], Anyarin Jirahiranpat[1], Suthicha Deewai[1], Panya Mackawan[4,6], Rasana W. Sermswan[1], Nisana Namwat[1,2,3], Watcharin Loilome[1,2,3], Tueanjit Khampitak[1], Arporn Wangwiwatsin[1,2,3], Jutarop Phetcharaburanin[1,2,3,7,*]

[1]Department of Biochemistry, Faculty of Medicine, Khon Kaen University, Khon Kaen, 40002, Thailand
[2]Khon Kaen University International Phenome Laboratory, Khon Kaen, 40002, Thailand
[3]Cholangiocarcinoma Research Institute, Khon Kaen University, Khon Kaen, 40002, Thailand
[4]Department of Entomology and Plant Pathology, Faculty of Agriculture, Khon Kaen University, Khon Kaen, 40002, Thailand
[5] Department of Microbiology, Faculty of Medicine, Khon Kaen University, Khon Kaen, 40002, Thailand
[6]Research and Development Center, Betagro Group, XXX
[7]Center of Excellence for Innovation in Chemistry, Faculty of Science, Khon Kaen University, Khon Kaen, 40002, Thailand

[*]Corresponding Author:
Jutarop Phetcharaburanin
Department of Biochemistry
Faculty of Medicine, Khon Kaen University
123 Mittraphab Road, Khon Kaen, 40002,
Thailand
jutarop@kku.ac.th

Table of Contents

Table of Contents………………………………………………………………… 2
Table of Figures……………………………………………………………….... 3
Table of Tables………………………………………………………………… 4
Figure S1……………………………………………………………………… 5
Table S1……………………………………………………………………….... 6
Table S2……………………………………………………………………….... 7
Table S3……………………………………………………………………….... 7
Table S4……………………………………………………………………….... 8
Table S5……………………………………………………………………….... 9
Table S6……………………………………………………………………….... 9
Table S7……………………………………………………………………….... 10
Table S8……………………………………………………………………….... 11

Table of Figures

Figure S1………………………………………………………………………………. 5

Table of Tables

Table S1…………………………………………………………………………... 6
Table S2…………………………………………………………………………... 7
Table S3…………………………………………………………………………... 7
Table S4…………………………………………………………………………... 8
Table S5…………………………………………………………………………... 9
Table S6…………………………………………………………………………... 9
Table S7…………………………………………………………………………... 10
Table S8…………………………………………………………………………... 11

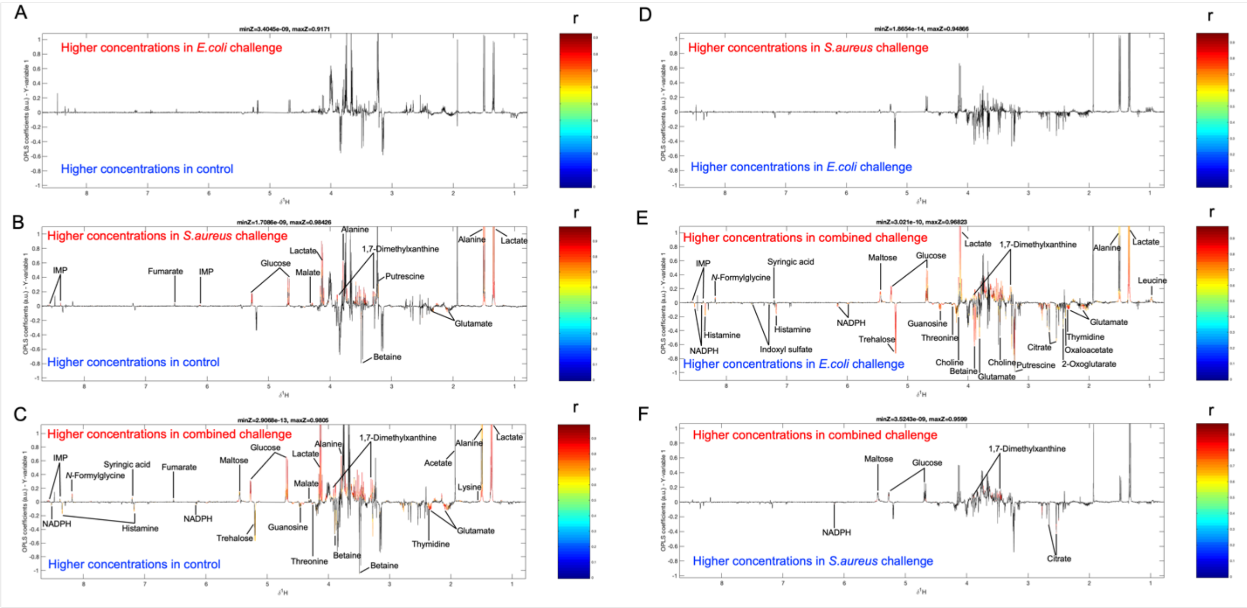

Figure S1. O-PLS-DA loading plots

O-PLS-DA loading plots of Control *vs.* Single *E. coli* challenge (A), Control *vs.* Single *S. aureus* challenge (B), Control *vs.* Combined challenge (C), Single *E. coli* challenge *vs.* Single *S. aureus* challenge (D), Single *E. coli* challenge *vs.* Combined challenge (E) and Single *S. aureus* challenge *vs.* Combined challenge (F). Resonances highlighted in red are significant metabolites after Benjamini-Hochberg correction (p-value < 0.05). Colour spectrum represents O-PLS-DA correlation coefficients (r).

Table S1. List of identified metabolites

No.	Metabolite	Chemical shift
1	Isoleucine	0.94 (t); 1.00 (d); 1.26 (m); 1.48 (m); 1.98 (m); 3.68 (d)
2	Leucine	0.96 (t); 1.71 (m); 3.73 (t)
3	Valine	0.99 (d); 1.04 (d); 2.28 (d); 3.62 (d)
4	Ethanol	1.19 (t); 3.60 (q)
5	Lactate	1.33 (d); 4.12 (q)
6	Threonine	1.36 (d); 3.59 (d); 4.26 (m)
7	Alanine	1.49 (d); 3.79 (q)
8	Acetate	1.93 (s)
9	Lysine	1.55 (m); 1.73 (q); 1.91 (m); 3.14 (t); 3.76 (t)
10	Glutamate	2.13 (m); 2.32 (dt); 3.80 (t)
11	Thymidine	1.89 (d); 2.37 (m); 3.76 (dd); 3.83 (dd); 4.01 (m); 4.50 (q); 6.29 (t); 7.63 (d)
12	Oxaloacetate	2.38 (s)
13	Succinate	2.41 (s)
14	2-Oxoglutarate	2.43 (t); 3.00 (t)
15	Carnitine	2.45 (dd); 3.23 (s); 3.43 (m)
16	Pyroglutamate	2.02 (m); 2.39 (m); 2.49 (m); 4.19 (q)
17	Citrate	2.54 (d); 2.66 (d)
18	Putrescine	1.73 (bs); 3.23 (bs)
19	Choline	3.36 (s); 3.47 (m); 4.17 (m)
20	Betaine	3.49 (s); 3.89 (s)
21	Malate	2.39 (dd); 2.69 (dd); 4.31 (dd)
22	Guanosine	4.19 (q); 4.45 (dd); 5.94 (d); 6.11 (d); 8.16 (s)
23	Glucose	3.40 (t); 3.53 (dd); 3.71 (t); 3.73 (dd); 3.83 (m); 5.27 (d)
24	Glucose-6-phosphate	3.28 (m); 3.50 (m); 3.56 (m); 3.72 (t); 3.88 (m); 3.99 (m); 4.64 (d); 5.23 (d)
25	Trehalose	3.45 (t); 3.64 (dd); 3.76 (dd); 3.81 (m); 3.85 (m); 5.19 (d)
26	Maltose	3.27 (m); 3.41 (t); 3.57 (m); 3.63 (m); 3.68 (m); 3.76 (m); 3.80 (m); 3.85 (m); 3.91 (m); 3.96 (m); 4.65 (d); 5.23 (d); 5.44 (d)
27	NADPH	2.76 (dd); 2.86 (t); 4.04 (m); 4.16 (m); 4.26 (m); 4.59 (t); 4.74 (d); 4.81 (m); 4.97 (m); 6.00 (dd); 6.15 (d); 8.30 (s); 8.52 (s)
28	Fumarate	6.53 (s)
29	Tyramine	2.92 (t); 3.23 (t); 6.93 (d); 7.20 (d)
30	Histamine	3.05 (t); 3.31 (t); 7.18 (s); 8.35 (d)
31	Indoxyl sulfate	7.22 (dd); 7.29 (dd); 7.38 (s); 7.56 (d); 7.73 (d)

32	Phenylalanine	3.13 (dd); 3.28 (dd); 4.00 (dd); 7.42 (m)
33	Dimethylxanthine	3.30 (s); 3.92 (s); 7.85 (s)
34	Formate	8.48 (s)
35	Syringic acid	3.90 (s); 7.20 (s);
36	N-Formylglycine	3.91 (d); 8.18 (s)
37	IMP	4.00 (m); 4.36 (m); 4.50 (m); 6.11 (d); 8.38 (s); 8.57 (s)

Key: s, singlet; bs, broad singlet; d, doublet; t, triplet; m, multiplet; q, quadruplet; dd, double doublet. Abbreviation: NADPH, nicotinamide adenine dinucleotide phosphate; IMP, inosine monophosphate.

Table S2. Multivariate statistical models

**Model**	**Cross-validated PCA**			**Cross-validated O-PLS-DA**			
	**R^2**		**Q^2**	**R^2X**	**R^2Y**	**Q^2Y**	**Permutation *p*-value**
	**PC1**	**PC2**					
All classes	35.3%	23.0%	0.61				
Control *vs.* Single *E. coli* challenge	37.9%	23.5%	0.45	54.7%	92.8%	0.80	0.01
Control *vs.* Single *S. aureus* challenge	39.4%	22.8%	0.53	54.0%	98.1%	0.91	0.01
Control *vs.* Combined challenge	50.3%	16.0%	0.64	65.2%	99.6%	0.67	0.01
Single *E. coli* challenge *vs.* Single *S. aureus* challenge	45.8%	26.1%	0.59	68.3%	89.5%	0.70	0.01
Single *E. coli* challenge *vs.* Combined challenge	43.7%	30.6%	0.55	54.7%	98.5%	0.91	0.01
Single *S. aureus vs.* Combined challenge	36.1%	22.7%	0.42	54.9%	94.7%	0.71	0.01

Table S3. Metabolic pathway analysis between control *vs.* single *E. coli* challenge

**Metabolic pathway**	**Total Cmpd**	**Hits**	**Raw *p***	**-LOG(*p*)**	**Holm adjust**	**FDR**	**Impact**
Lysine degradation	21	1	0.00025041	3.6014	0.0062601	0.0031301	0
Biotin metabolism	10	1	0.00025041	3.6014	0.0062601	0.0031301	0
Arginine and proline metabolism	31	2	0.032101	1.4935	0.73833	0.20116	0.18713
Glutathione metabolism	26	3	0.032185	1.4923	0.73833	0.20116	0.05891
Valine, leucine and isoleucine degradation	38	1	0.049045	1.3094	1	0.21676	0
Aminoacyl-tRNA biosynthesis	48	5	0.060283	1.2198	1	0.21676	0
Tyrosine metabolism	33	1	0.060694	1.2169	1	0.21676	0.04121
Caffeine metabolism	10	1	0.092764	1.0326	1	0.28989	0
Alanine, aspartate and glutamate metabolism	23	5	0.12536	0.90185	1	0.34821	0.43582
Valine, leucine and isoleucine biosynthesis	8	2	0.166	0.77989	1	0.41501	0
Pyruvate metabolism	22	5	0.26629	0.57465	1	0.54644	0.12213
Glycolysis / Gluconeogenesis	26	4	0.26661	0.57412	1	0.54644	0.02889
Pyrimidine metabolism	40	1	0.28415	0.54645	1	0.54644	0
Glycine, serine and threonine metabolism	30	3	0.37399	0.42714	1	0.587	0.03846
Porphyrin and chlorophyll metabolism	24	1	0.37568	0.42518	1	0.587	0
Nitrogen metabolism	5	1	0.37568	0.42518	1	0.587	0
Glycerophospholipid metabolism	32	1	0.40073	0.39714	1	0.58932	0.05419
Arginine biosynthesis	12	3	0.60183	0.22053	1	0.73715	0.05714

D-Glutamine and D-glutamate metabolism	5	2	0.61438	0.21156	1	0.73715	1
Butanoate metabolism	14	2	0.61438	0.21156	1	0.73715	0
Citrate cycle (TCA cycle)	20	5	0.6192	0.20817	1	0.73715	0.34069
Histidine metabolism	9	1	0.70794	0.15	1	0.77719	0
Glyoxylate and dicarboxylate metabolism	24	5	0.71757	0.14414	1	0.77719	0.0875
Purine metabolism	63	2	0.7461	0.1272	1	0.77719	0.10929
Starch and sucrose metabolism	14	1	0.81266	0.090092	1	0.81266	0.01294

Total Cmpd, the total number of compounds in the pathway; Hits, the matched metabolites uploaded in MetaboAnalyst; Raw p, the original *p*-value calculated from the enrichment analysis; Holm adjust, *p*-value adjusted by Holm-Bonferroni method; FDR, the *p*-value adjusted by the false discovery rate; Impact, the pathway impact value calculated from pathway topology analysis.

Table S4. Metabolic pathway analysis between control *vs.* single *S. aureus* challenge

Metabolic pathway	Total Cmpd	Hits	Raw p	**-LOG(*p*)**	Holm adjust	FDR	Impact
Glycolysis / Gluconeogenesis	26	4	1.12E-05	4.9503	0.00028031	0.00014071	0.02889
Pyruvate metabolism	22	5	1.13E-05	4.9486	0.00028031	0.00014071	0.12213
Alanine, aspartate and glutamate metabolism	23	5	2.24E-05	4.6491	0.00051593	0.00017009	0.43582
Aminoacyl-tRNA biosynthesis	48	5	2.72E-05	4.5652	0.00059873	0.00017009	0
Caffeine metabolism	10	1	0.00074494	3.1279	0.015644	0.0037247	0
Tyrosine metabolism	33	1	0.0026175	2.5821	0.052349	0.010906	0.04121
Glyoxylate and dicarboxylate metabolism	24	5	0.0050157	2.2997	0.095298	0.017913	0.0875
Arginine and proline metabolism	31	2	0.028676	1.5425	0.51616	0.072879	0.18713
Glutathione metabolism	26	3	0.028705	1.542	0.51616	0.072879	0.05891
Valine, leucine and isoleucine biosynthesis	8	2	0.029152	1.5353	0.51616	0.072879	0
Starch and sucrose metabolism	14	1	0.10493	0.9791	1	0.23848	0.01294
Pyrimidine metabolism	40	1	0.15989	0.79617	1	0.33311	0
Glycine, serine and threonine metabolism	30	3	0.19294	0.71457	1	0.37104	0.03846
Histidine metabolism	9	1	0.2604	0.58437	1	0.46499	0
Purine metabolism	63	2	0.28503	0.54511	1	0.47505	0.10929
Glycerophospholipid metabolism	32	1	0.33407	0.47617	1	0.50571	0.05419
Lysine degradation	21	1	0.37951	0.42078	1	0.50571	0
Biotin metabolism	10	1	0.37951	0.42078	1	0.50571	0
Porphyrin and chlorophyll metabolism	24	1	0.40457	0.39301	1	0.50571	0
Nitrogen metabolism	5	1	0.40457	0.39301	1	0.50571	0
Arginine biosynthesis	12	3	0.68549	0.164	1	0.78918	0.05714
D-Glutamine and D-glutamate metabolism	5	2	0.7394	0.13112	1	0.78918	1
Butanoate metabolism	14	2	0.7394	0.13112	1	0.78918	0
Citrate cycle (TCA cycle)	20	5	0.75761	0.12055	1	0.78918	0.34069
Valine, leucine and isoleucine degradation	38	1	0.99695	0.0013286	1	0.99695	0

Total Cmpd, the total number of compounds in the pathway; Hits, the matched metabolites uploaded in MetaboAnalyst; Raw p, the original *p*-value calculated from the enrichment analysis; Holm adjust, *p*-value adjusted by Holm-Bonferroni method; FDR, the *p*-value adjusted by the false discovery rate; Impact, the pathway impact value calculated from pathway topology analysis.

Table S5. Metabolic pathway analysis between control *vs.* Combined challenge

Metabolic pathway	Total Cmpd	Hits	Raw *p*	-LOG(*p*)	Holm adjust	FDR	Impact
Glycolysis / Gluconeogenesis	26	4	0.00027381	3.5626	0.0068452	0.0034348	0.02889
Pyruvate metabolism	22	5	0.00027479	3.561	0.0068452	0.0034348	0.12213
Caffeine metabolism	10	1	0.0016832	2.7739	0.038714	0.0077829	0
Glyoxylate and dicarboxylate metabolism	24	5	0.0017747	2.7509	0.039044	0.0077829	0.0875
Aminoacyl-tRNA biosynthesis	48	5	0.0017897	2.7472	0.039044	0.0077829	0
Alanine, aspartate and glutamate metabolism	23	5	0.0018679	2.7286	0.039044	0.0077829	0.43582
Tyrosine metabolism	33	1	0.0053317	2.2731	0.1013	0.019042	0.04121
Histidine metabolism	9	1	0.016767	1.7755	0.30181	0.052398	0
Starch and sucrose metabolism	14	1	0.020148	1.6958	0.34251	0.055966	0.01294
Purine metabolism	63	2	0.023766	1.624	0.38025	0.059415	0.10929
Valine, leucine and isoleucine biosynthesis	8	2	0.031511	1.5015	0.47267	0.071617	0
Pyrimidine metabolism	40	1	0.11354	0.94486	1	0.23654	0
Glutathione metabolism	26	3	0.13762	0.86132	1	0.24599	0.05891
Arginine and proline metabolism	31	2	0.13775	0.8609	1	0.24599	0.18713
Lysine degradation	21	1	0.16159	0.79159	1	0.25248	0
Biotin metabolism	10	1	0.16159	0.79159	1	0.25248	0
Valine, leucine and isoleucine degradation	38	1	0.299	0.52433	1	0.4397	0
Citrate cycle (TCA cycle)	20	5	0.42648	0.3701	1	0.59233	0.34069
Glycine, serine and threonine metabolism	30	3	0.47103	0.32695	1	0.60419	0.03846
Porphyrin and chlorophyll metabolism	24	1	0.50752	0.29455	1	0.60419	0
Nitrogen metabolism	5	1	0.50752	0.29455	1	0.60419	0
Arginine biosynthesis	12	3	0.70391	0.15248	1	0.76243	0.05714
D-Glutamine and D-glutamate metabolism	5	2	0.73193	0.13553	1	0.76243	1
Butanoate metabolism	14	2	0.73193	0.13553	1	0.76243	0
Glycerophospholipid metabolism	32	1	0.9945	0.0023959	1	0.9945	0.05419

Total Cmpd, the total number of compounds in the pathway; Hits, the matched metabolites uploaded in MetaboAnalyst; Raw p, the original *p*-value calculated from the enrichment analysis; Holm adjust, *p*-value adjusted by Holm-Bonferroni method; FDR, the *p*-value adjusted by the false discovery rate; Impact, the pathway impact value calculated from pathway topology analysis.

Table S6. Metabolic pathway analysis between single *E. coli* challenge *vs.* single *S. aureus* challenge

Metabolic pathway	Total Cmpd	Hits	Raw *p*	-LOG(*p*)	Holm adjust	FDR	Impact
Glycolysis / Gluconeogenesis	26	4	0.00085117	3.07	0.021279	0.0083432	0.02889
Pyruvate metabolism	22	5	0.00085118	3.07	0.021279	0.0083432	0.12213
Aminoacyl-tRNA biosynthesis	48	5	0.001271	2.8959	0.029233	0.0083432	0
Alanine, aspartate and glutamate metabolism	23	5	0.0013349	2.8745	0.029368	0.0083432	0.43582
Starch and sucrose metabolism	14	1	0.0060048	2.2215	0.1261	0.030024	0.01294
Valine, leucine and isoleucine degradation	38	1	0.012431	1.9055	0.24861	0.042987	0

Lysine degradation	21	1	0.013756	1.8615	0.26136	0.042987	0
Biotin metabolism	10	1	0.013756	1.8615	0.26136	0.042987	0
Glyoxylate and dicarboxylate metabolism	24	5	0.016149	1.7918	0.27454	0.044859	0.0875
Caffeine metabolism	10	1	0.02976	1.5264	0.47616	0.0744	0
Histidine metabolism	9	1	0.033284	1.4778	0.49926	0.075646	0
Valine, leucine and isoleucine biosynthesis	8	2	0.090716	1.0423	1	0.18899	0
Purine metabolism	63	2	0.14755	0.83105	1	0.28376	0.10929
Glycine, serine and threonine metabolism	30	3	0.44391	0.3527	1	0.78494	0.03846
Tyrosine metabolism	33	1	0.47097	0.32701	1	0.78494	0.04121
Citrate cycle (TCA cycle)	20	5	0.63889	0.19457	1	0.85117	0.34069
Pyrimidine metabolism	40	1	0.6583	0.18158	1	0.85117	0
Arginine biosynthesis	12	3	0.73545	0.13345	1	0.85117	0.05714
D-Glutamine and D-glutamate metabolism	5	2	0.73637	0.1329	1	0.85117	1
Butanoate metabolism	14	2	0.73637	0.1329	1	0.85117	0
Porphyrin and chlorophyll metabolism	24	1	0.74903	0.1255	1	0.85117	0
Nitrogen metabolism	5	1	0.74903	0.1255	1	0.85117	0
Glutathione metabolism	26	3	0.81656	0.088012	1	0.8548	0.05891
Arginine and proline metabolism	31	2	0.82061	0.085863	1	0.8548	0.18713
Glycerophospholipid metabolism	32	1	0.95641	0.019358	1	0.95641	0.05419

Total Cmpd, the total number of compounds in the pathway; Hits, the matched metabolites uploaded in MetaboAnalyst; Raw p, the original *p*-value calculated from the enrichment analysis; Holm adjust, *p*-value adjusted by Holm-Bonferroni method; FDR, the *p*-value adjusted by the false discovery rate; Impact, the pathway impact value calculated from pathway topology analysis.

Table S7. Metabolic pathway analysis between single *E. coli* challenge *vs.* Combined challenge

Metabolic pathway	Total Cmpd	Hits	Raw *p*	-LOG(*p*)	Holm adjust	FDR	Impact
Starch and sucrose metabolism	14	1	0.00081752	3.0875	0.020438	0.0082283	0.01294
Glycolysis / Gluconeogenesis	26	4	0.0009857	3.0063	0.023657	0.0082283	0.02889
Pyruvate metabolism	22	5	0.0009874	3.0055	0.023657	0.0082283	0.12213
Histidine metabolism	9	1	0.0015746	2.8028	0.03464	0.0098409	0
Glyoxylate and dicarboxylate metabolism	24	5	0.0028138	2.5507	0.05909	0.014069	0.0875
Purine metabolism	63	2	0.0074225	2.1295	0.14845	0.027655	0.10929
Valine, leucine and isoleucine degradation	38	1	0.0077434	2.1111	0.14845	0.027655	0
Caffeine metabolism	10	1	0.0098005	2.0088	0.17641	0.02908	0
Aminoacyl-tRNA biosynthesis	48	5	0.010469	1.9801	0.17797	0.02908	0
Alanine, aspartate and glutamate metabolism	23	5	0.011718	1.9311	0.18749	0.029295	0.43582
Lysine degradation	21	1	0.04016	1.3962	0.6024	0.083667	0
Biotin metabolism	10	1	0.04016	1.3962	0.6024	0.083667	0
Valine, leucine and isoleucine biosynthesis	8	2	0.092337	1.0346	1	0.17757	0
Citrate cycle (TCA cycle)	20	5	0.15089	0.82135	1	0.26944	0.34069
Tyrosine metabolism	33	1	0.35042	0.45541	1	0.54493	0.04121
Glycine, serine and threonine metabolism	30	3	0.36221	0.44104	1	0.54493	0.03846

Pyrimidine metabolism	40	1	0.37055	0.43115	1	0.54493	0
Glycerophospholipid metabolism	32	1	0.44321	0.35339	1	0.57625	0.05419
Glutathione metabolism	26	3	0.45652	0.34054	1	0.57625	0.05891
Arginine and proline metabolism	31	2	0.461	0.3363	1	0.57625	0.18713
Arginine biosynthesis	12	3	0.77753	0.10928	1	0.84645	0.05714
D-Glutamine and D-glutamate metabolism	5	2	0.77873	0.10861	1	0.84645	1
Butanoate metabolism	14	2	0.77873	0.10861	1	0.84645	0
Porphyrin and chlorophyll metabolism	24	1	0.99108	0.0038918	1	0.99108	0
Nitrogen metabolism	5	1	0.99108	0.0038918	1	0.99108	0

Total Cmpd, the total number of compounds in the pathway; Hits, the matched metabolites uploaded in MetaboAnalyst; Raw p, the original *p*-value calculated from the enrichment analysis; Holm adjust, *p*-value adjusted by Holm-Bonferroni method; FDR, the *p*-value adjusted by the false discovery rate; Impact, the pathway impact value calculated from pathway topology analysis.

Table S8. Metabolic pathway analysis between single *S. aureus* challenge *vs.* Combined challenge

Metabolic pathway	Total Cmpd	Hits	Raw *p*	-LOG(*p*)	Holm adjust	FDR	Impact
Histidine metabolism	9	1	0.013304	1.876	0.33261	0.18509	0
Starch and sucrose metabolism	14	1	0.014807	1.8295	0.35537	0.18509	0.01294
Purine metabolism	63	2	0.033659	1.4729	0.77417	0.19337	0.10929
Glycolysis / Gluconeogenesis	26	4	0.038486	1.4147	0.84669	0.19337	0.02889
Pyruvate metabolism	22	5	0.038675	1.4126	0.84669	0.19337	0.12213
Glyoxylate and dicarboxylate metabolism	24	5	0.087032	1.0603	1	0.32993	0.0875
Caffeine metabolism	10	1	0.092381	1.0344	1	0.32993	0
Lysine degradation	21	1	0.12289	0.91047	1	0.34137	0
Biotin metabolism	10	1	0.12289	0.91047	1	0.34137	0
Valine, leucine and isoleucine degradation	38	1	0.20068	0.69749	1	0.4752	0
Citrate cycle (TCA cycle)	20	5	0.20909	0.67967	1	0.4752	0.34069
Aminoacyl-tRNA biosynthesis	48	5	0.29198	0.53465	1	0.59592	0
Alanine, aspartate and glutamate metabolism	23	5	0.33162	0.47935	1	0.59592	0.43582
Glutathione metabolism	26	3	0.36257	0.44061	1	0.59592	0.05891
Arginine and proline metabolism	31	2	0.36317	0.43989	1	0.59592	0.18713
Glycerophospholipid metabolism	32	1	0.38638	0.41298	1	0.59592	0.05419
Glycine, serine and threonine metabolism	30	3	0.40523	0.3923	1	0.59592	0.03846
Pyrimidine metabolism	40	1	0.51817	0.28553	1	0.71968	0
Valine, leucine and isoleucine biosynthesis	8	2	0.64663	0.18935	1	0.81178	0
Tyrosine metabolism	33	1	0.64943	0.18747	1	0.81178	0.04121
Porphyrin and chlorophyll metabolism	24	1	0.821	0.085659	1	0.92047	0
Nitrogen metabolism	5	1	0.821	0.085659	1	0.92047	0
Arginine biosynthesis	12	3	0.91826	0.037036	1	0.92047	0.05714

D-Glutamine and D-glutamate metabolism	5	2	0.92047	0.035991	1	0.92047	1
Butanoate metabolism	14	2	0.92047	0.035991	1	0.92047	0

Total Cmpd, the total number of compounds in the pathway; Hits, the matched metabolites uploaded in MetaboAnalyst; Raw p, the original *p*-value calculated from the enrichment analysis; Holm adjust, *p*-value adjusted by Holm-Bonferroni method; FDR, the *p*-value adjusted by the false discovery rate; Impact, the pathway impact value calculated from pathway topology analysis.
